# Functional Consequences of Calcium Influx Promoted by Bacterial Pore-Forming Toxins

**DOI:** 10.3390/toxins10100387

**Published:** 2018-09-25

**Authors:** Stéphanie Bouillot, Emeline Reboud, Philippe Huber

**Affiliations:** Université Grenoble Alpes, CNRS ERL5261, CEA BIG-BCI, INSERM UMR1036, Grenoble 38054, France; stephanie.bouillot@cea.fr (S.B.); emeline.reboud@gmail.com (E.R.)

**Keywords:** host–pathogen interaction, bacterial virulence factor, cell death, signal transduction, ion flux

## Abstract

Bacterial pore-forming toxins induce a rapid and massive increase in cytosolic Ca^2+^ concentration due to the formation of pores in the plasma membrane and/or activation of Ca^2+^-channels. As Ca^2+^ is an essential messenger in cellular signaling, a sustained increase in Ca^2+^ concentration has dramatic consequences on cellular behavior, eventually leading to cell death. However, host cells have adapted mechanisms to protect against Ca^2+^ intoxication, such as Ca^2+^ efflux and membrane repair. The final outcome depends upon the nature and concentration of the toxin and on the cell type. This review highlights the repercussions of Ca^2+^ overload on the induction of cell death, repair mechanisms, cellular adhesive properties, and the inflammatory response.

## 1. Introduction

Bacterial pore-forming toxins (PFTs) are the most frequently encountered virulence factors among bacterial pathogens [1,2,3,4]. They are secreted in the extracellular milieu from Gram-negative and Gram-positive bacteria by various bacterial secretion systems. It is generally accepted that PFTs need a specific cellular receptor to bind to the host cell and to form a pore, hence preventing bacterial self-toxicity. Several cellular receptors for PFTs have been identified, so far, from membrane proteins specific to one or more cell types of multicellular organisms, to lipids like cholesterol or sphingomyelin, which are frequently present in vertebrate tissues [3,5]. These two lipids are concentrated in specialized membrane domains called lipid rafts, which are also the site of localization of specific membrane proteins or receptors. As a result, several PFTs are guided toward these rafts.

Once bound to a target cell receptor, PFTs form a pore in the plasma membrane, using a complex multistep process. The first step is toxin oligomerization. Oligomerization may occur once the toxins are inserted into the membrane or otherwise bound to the cell’s surface, forming a prepore. The prepore then inserts into the plasma membrane, forming a ring-shaped pore, which alters the target cell’s integrity [3]. Pore formation may have dramatic consequences for the host cell, unless it can mount a process to eliminate the membrane domain containing the pore. Importantly, mechanisms dependent on the toxins, but independent of pore formation, have also been reported. They involve receptor activation and downstream signaling that can also alter Ca^2+^ concentration within host cells. The various scenarios described after PFT intoxication depend mainly on PFT identity and its local concentration, but also on the host cell type (e.g., immune vs nonimmune cells, epithelium vs. endothelium). For all these effects, Ca^2+^ is a central player in PFT-induced toxicity and downregulation of its cytosolic concentration is critical for cell fate.

## 2. How Do PFTs Increase Intracellular Ca^2+^?

**Figure 1:**
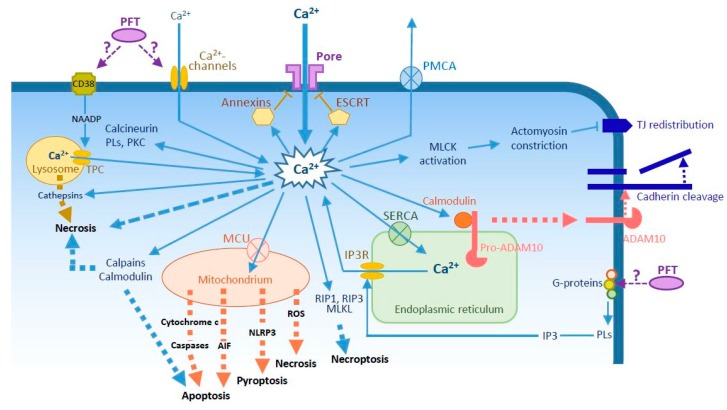
Potential Ca^2+^ circuitry induced by pore-forming toxins (PFTs) and main reported effects of sustained Ca^2+^ elevation. Increased cytosolic Ca^2+^ concentrations can be induced by passive flow through the pore and/or activation of Ca^2+^-channels either in the plasma membrane, in the endoplasmic reticulum (ER) (IP3R, via G-proteins-PLs-IP3 pathway), or in the lysosome (TPC, via CD38-NAADP pathway). Ca^2+^ pumps in the plasma membrane (PMCA), the ER (SERCA) and the mitochondria (MCU) are employed to maintain low levels of cytosolic Ca^2+^. Ca^2+^ binds and activates members of annexin family to promote pore endocytosis, or the ESCRT complex for microvesicle secretion. Ca^2+^ interacts also with calmodulin, which detaches from pro-ADAM10, allowing its maturation and export to the plasma membrane where it cleaves cadherins. Ca^2+^ activates a number of proteins, including MLCK, which promotes actomyosin constriction and TJ protein redistribution from the junction. Ca^2+^ intoxication activates several cell death pathways: (i) necrosis can be induced by osmotic lysis, by activated calmodulin and calpains, by release of cathepsins from lysosomes or ROS from mitochondria; (ii) apoptosis by release of AIF and cytochrome c from mitochondria or by activated calpains and calmodulin; (iii) NLRP3-dependent pyroptosis from mitochondrial signals; and (iv) necroptosis, by activation of RIP1, RIP3, and MLKL. Abbreviations: AIF, apoptosis-inducing factor; ESCRT, endosomal sorting complex required for transport; IP3R, inositol triphosphate receptor; MLCK, myosin light chain-kinase; MCU, mitochondrial Ca^2+^ uniporter; MLKL, mixed-lineage kinase domain-like protein; NAADP, nicotinic acid adenine dinucleotide phosphate; PKC, protein kinase C; PL, phospholipase; PMCA, plasma membrane Ca^2+^-ATPase; RIP, receptor interacting protein; ROS, reactive oxygen species; SERCA, sarco/endoplasmic reticulum Ca^2+^-ATPase; TJ, tight junction; TPC, two-pore channel.

In general, one of the immediate consequences of PFT insertion into the plasma membrane is ion exchange between the extracellular environment and the cytosol through the open pore. For intracellular bacteria, pore formation and ion flux can also occur within the phagosome [6]. Other mechanisms have been described, such as the activation of endogenous ion channels, either located in the plasma membrane or in organelles accumulating Ca^2+^, like the endoplasmic reticulum (ER) and lysosomes (Figure 1) [7,8,9,10,11,12,13,14,15].

Pores or ion channels can exchange a range of ion types, but most previous studies have concentrated on Ca^2+^ and K^+^ flux, because they are known to have important functional consequences. These ions passively flow through the open pores as a result of the concentration gradients between the external milieu and the cytosol: millimolar Ca^2+^ concentrations are reported in animal tissues and fluids, and 10,000-fold lower concentrations are found in the cytosol [16]; in contrast, K^+^ concentration is in the millimolar range in animal fluids, and 130 mM in the cytosol [17]. Hence, pore formation triggers passive K^+^ efflux and Ca^2+^ influx. Although passive ion flow is the rule (see Table 1), at least one PFT, the *Vibrio cholerae* cytolysin, creates a pore that is too narrow to allow passage of Ca^2+^ [18]. The related PFT, phobalysin, from *Photobacterium dameselae*, is large enough to allow Ca^2+^ flux [18]. A single mutation in *V. cholerae* cytolysin, rendering the channel domain similar to that of phobalysin and enlarging the pore, makes Ca^2+^ influx possible and modifies the host response to pore formation. Interestingly, K^+^ can flow out through the unmodified cytolysin, suggesting that some ion selection may exist in the pores created by PFTs.

Importantly, K^+^ efflux is well known to induce several host cell alterations, including the activation of the NLRP3 inflammasome and p38 MAP kinase. The functional implications of K^+^ efflux have been reviewed in details elsewhere [19,20,21,22]. However, it has also been reported that mitochondrial Ca^2+^ elevation, a secondary effect of cytosolic Ca^2+^ rise, can promote NLRP3 activation [23,24], hence positioning Ca^2+^ as another potent initiator of inflammasome activation.

Ca^2+^ influx through the pore is usually massive because of the very steep concentration gradient between the extracellular and cytosolic compartments. Therefore, Ca^2+^ entry usually displays monophasic kinetics, eventually followed by a sudden drop if the cell bursts, delivering its content in the extracellular milieu (biphasic kinetics). However, multiphasic kinetics may also be observed, either because of rapid opening/closing of the pore or because forming pores are progressively eliminated by the host cell’s repair mechanisms (see below and references in Table 1).

Ca^2+^ oscillations have also been described for some PFTs when Ca^2+^ channels are activated (Table 1). Release of Ca^2+^ from internal stores has been reported for several PFTs using different pathways. In addition to formation of Ca^2+^-permeable pores, some PFTs, like aerolysin from *Aeromonas hydrophila*, streptolysin O (SLO) from *Streptococcus pyogenes*, and *Staphylococcus aureus* hemolysin A (Hla) [12], induce the release of Ca^2+^ from the ER by two different mechanisms successively: (i) a transient Ca^2+^ release from inositol (1,4,5)P3-sensitive stores which involves G-proteins and phospholipase C, and (ii) a delayed and sustained release, the activation mechanisms of which remain to be determined [12].

Similarly, listeriolysin (LLO) from *Listeria monocytogenes* induces Ca^2+^ release from the ER via the G protein-phospholipase C-inositol (1,4,5)P3 pathway, as well as a second wave of Ca^2+^ release involving damage to intracellular stores (ER and lysosomes) [9]. The mechanism leading to organelle perforation is unknown but seems to be Ca^2+^–independent. Interestingly, organelle damage is reversible and does not result in cell death. This is an unconventional but efficient way to deliver Ca^2+^ in the cytosol, because of the high Ca^2+^ content of the ER.

Finally, *Pasteurella haemolytica* leukotoxin (LKT) induces increased cytosolic Ca^2+^ by activating voltage-gated channels in the plasma membrane via a G-protein-coupled mechanism involving activation of phospholipases A2 and C [7,10],

The mechanism of G-protein activation by PFTs remains undetermined. It is tempting to speculate that PFTs interact with a G-protein coupled receptor at the cell surface, which is the common way for G-protein activation. Alternatively, the transmembrane pore formed by PFTs may interact directly with G-proteins in the cytosol, without the need of a specific receptor.

Other examples of PFTs with specific modes of action are *S. aureus* leukotoxins (γ-hemolysin, Hlg, and Panton–Valentine leukocidin, PVL), which increase Ca^2+^ levels by triggering its release from lysosomes followed by a second release from endoplasmic reticulum. This in turn stimulates the activation of store-operated Ca^2+^-channels in the plasma membrane, a process normally used when intracellular organelles are discharged of Ca^2+^ [11]. The initial signal linking leukotoxin binding to acidic stores is the activation of the ADP-ribosyl cyclase CD38 [11]. CD38 is a membrane receptor and a nicotinic acid adenine dinucleotide phosphate (NAADP) synthase required for coupling receptor activation to NAADP-mediated Ca^2+^ release from lysosomal stores through the two-pore Ca^2+^ channels [25,26].

Finally, *Bordetella pertussis* ACT, through its adenylate cyclase properties, activates non-voltage-dependent Ca^2+^ channels with L-type characteristics. This process involves ACT-induced cAMP production and subsequent protein kinase A activation [13].

Thus, although passive influx through the pore is the most widespread mode of Ca^2+^ entry exploited by bacterial PFTs, several toxins can also use other pathways to increase cytosolic Ca^2+^ concentration. It is yet unknown whether Ca^2+^ channel opening occurs before or simultaneously with pore formation.

In general, Ca^2+^ is maintained at low levels in the cytosol because it is an important second messenger activating several signaling pathways. Ca^2+^ can interact with and activate a number of cytoplasmic proteins [16] that are potential sensors of the presence of PFT pores. Effective activation by PFTs have been reported for calmodulin [34,48], calpains [34,37,61], protein kinase C (PKC) [43], phospholipases [50], and calcineurin [54]. Sustained activation of calpain, PKC, and calcineurin pathways leads to cell death. Therefore, several mechanisms are employed by the cell to maintain low concentrations of cytosolic Ca^2+^. Extrusion of cytosolic Ca^2+^ can be carried out by plasma membrane pumps (the plasma membrane Ca^2+^-ATPase; PMCA) [62], and was reported for Hla [49]. Large amounts of Ca^2+^ can also be efficiently sequestered in the ER thanks to the sarco/endoplasmic reticulum Ca^2+^-ATPase (SERCA) [63] or into mitochondria via the mitochondrial Ca^2+^ uniporter (MCU) [64], which eventually leads to mitochondrial intoxication. However, massive Ca^2+^ entry rapidly overloads the cytosol and exceeds the capacity of internal stores and the capability of Ca^2+^ pumps. If no membrane repair mechanism is activated, ion imbalance eventually triggers osmotic rupture of the plasma membrane or cell death by another pathway (see below).

In the following sections, we will focus on the main consequences of sustained PFT-dependent increase in cytosolic Ca^2+^ reported in the literature. Other major effects of pore formation by PFTs, for which the link with Ca^2+^ has not been established, are not dealt with here.

## 3. Cell Repair Mechanisms

Host cells have adapted to PFT injury by creating several mechanisms to eliminate pores that efficiently combat the dramatic effects of cell perforation by PFTs. The repair mechanism used depends upon the nature and number of pores, and on the cell type. As for the Ca^2+^ exporting systems, when the repair mechanisms are overwhelmed, the cellular ion imbalance reaches a point-of-no-return and cells engage in an irreversible process of cell death. Several excellent reviews have recently been published on this topic [1,2,4,65,66]; here, we will simply summarize the main mechanisms used by the cell to repair its plasma membrane.

Membrane repair mechanisms have been studied for cholesterol-binding PFTs (SLO, *Streptococcus pneumoniae* pneumolysin (PLY), *Clostridium perfringens* perfringolysin (PFO), and *Streptococcus intermedius* intermedilysin (ILY)) forming large pores (30 nm in diameter) that are surprisingly more efficiently eliminated by host cells than small-pore forming toxins.

The primary mechanism of pore clearance involves the externalization of microvesicles (also called ectocytosis) containing PFT pores [33,67,68,69,70]. This can be performed via two different ways involving either (i) annexins, which migrate to the injured site, avidly bind to Ca^2+^ and interact with the plasma membrane; because of their fusogenic activity, annexins induce the formation of membrane folds that can be expelled; or (ii) the endosomal sorting complex required for transport (ESCRT) machinery, which drives microvesicle shedding [71,72].

The other process described is the endocytic model, whereby a portion of pore-containing membrane is internalized and then targeted to the lysosome for degradation [60,73]. This process seems to be restricted to the elimination of inactivated or monomeric toxins [33].

Both processes of membrane repair (ectocytosis or endocytosis) require RAB-5 and RAB-11, two important regulators of vesicle trafficking [74].

As mentioned above, PFTs forming large pores trigger repair mechanisms much more efficiently than small-pore toxins. The reasons for this difference in triggering capacity remain elusive. One obvious hypothesis would be that small pores do not promote a Ca^2+^ influx sufficient for repair mechanism activation, however this hypothesis is contradicted by Ca^2+^ imaging data showing massive influx when cells are incubated with small-pore forming toxins [48,49,50]. As PFTs inducing efficient membrane repair interact directly with lipids and stimulate blebbing at the prepore stage, it is possible that they are cleared before membrane damage. Conversely, PFTs interacting with proteinaceous receptors may not induce the intrinsic pathway and cells may only depend upon the endocytic repair mechanism. Other collateral factors may include the capacity of PFTs to induce Ca^2+^ channel opening or pore stability in the plasma membrane.

Interestingly, ACT controls the path and kinetics of endocytic removal of toxin pores in a K^+^-dependent manner [32]. As many PFT pores were reported to trigger K^+^ efflux, this repair mechanism may also be true for most PFT pores.

Other Ca^2+^-independent repair mechanisms have also been described, such as p38 and JNK MAP kinase activation [75,76] and activation of the unfolded protein response [77]. The contribution to and modes of action of these latter mechanisms in membrane repair remain undetermined.

## 4. Cell Death

If the repair mechanisms fail to remove PFT pores, the cells will eventually commit to Ca^2+^-dependent death programs (Table 2).

PFT-induced cell death is often reported as “osmotic lysis”, a type of necrosis involving cell dilation and membrane rupture due to excessive intracellular pressure [34,35,37,59]. As previously mentioned, this results from sustained ion flux causing osmotic imbalance due to the high concentration of macromolecules inside the cell [78]. However, the kinetics of PFT-mediated Ca^2+^ influx induces cell death that may be more related to Ca^2+^ toxicity than ion imbalance [30], and a number of PFTs trigger cell death by apoptosis, alone or in parallel with necrosis [29,34,59,61,79].

In some examples [29,34,59,79], low doses of PFTs can promote Ca^2+^-dependent apoptosis by eliciting the release of apoptosis-inducing factor (AIF) and cytochrome c, owing to Ca^2+^-induced opening of the mitochondrial permeability transition pore. Both proteins are known proapoptotic factors: AIF is translocated to the nucleus where it causes DNA degradation and chromatin condensation; cytochrome c activates the caspase cascade leading to DNA fragmentation [80]. Independently, calpain protease activation by Ca^2+^ can also trigger caspase-dependent or independent apoptosis as well as necrosis [78,81]. All three pathways (cytochrome c, AIF, and calpains) may be instrumental for PFTs to promote cell death of intoxicated cells. For example, *C. perfringens* enterotoxin (CPE) elicits apoptosis at low doses and necrosis at high doses, both pathways being caspase- and calpain-dependent [34]. In addition, calmodulin, a cytosolic protein with high affinity for Ca^2+^, is also involved in CPE-dependent apoptosis and necrosis processes [34]. The mechanism of calmodulin-induced cell death was not determined in this context, but recent work in breast cancer cells demonstrated that calmodulin can bind to death receptor-5 (DR5) in a Ca^2+^-dependent manner, which triggers apoptotic signaling [82]. This mechanism may also occur when cells are intoxicated with CPE.

Similarly, *C. perfringens* epsilon toxin (ET) stimulates the release of cytochrome c and mitochondrial–nuclear translocation of AIF, leading to chromatin condensation and nuclear shrinkage. However, ET fails to induce DNA fragmentation and thus to achieve apoptosis; cell death being then executed by necrosis [35]. A possible explanation is that the energy-dependent process of apoptosis is dissipated as a result of the loss of ATP through the pore, whereas necrosis can be completed as it requires no energy [35].

In the case of α-toxin from *C. septicum*, Ca^2+^ influx induces a mechanism of programmed necrosis involving calpain activation, release of cathepsins from lysosomes and increased reactive oxygen species (ROS) levels produced by deregulation of mitochondrial activity [37]. Thus, PFT-dependent necrosis may not just result from oncosis, but from damage of cellular organelles together with activation of cytosolic Ca^2+^-sensor proteins.

Finally, two PFTs, ShlA and PLY, can trigger Ca^2+^-dependent necroptosis in pneumocytes [83]. Necroptosis is a regulated cell death program leading to cell membrane rupture, and as such is considered as a form of necrosis [84]. In general, necroptosis is engaged by membrane receptors, tumor necrosis factor receptors (TNFRs) or Toll-like receptors (TLRs), and is followed by a cascade of signaling that occurs only when caspases are inactivated [84]. Signaling proteins include the receptor interacting proteins (RIP) 1 and 3, and the mixed-lineage kinase domain-like protein (MLKL), the executer of necroptosis. Gonzales-Juarbe et al. [83] showed that *Serratia marcescens* hemolysin (ShlA)- and PLY-induced necroptosis were independent of TNFR or TLR activation, but required RIP1, RIP3, and MLKL. This was confirmed in vivo in mice deficient either in RIP3 or in MLKL that exhibited increased survival when challenged with *S. marcescens*.

Taken together, PFTs can act on several pathways, probably in combination, to provoke cell death. It is noteworthy to point out the central role of mitochondria in this context. Still, much work has to be done to fully elucidate this critical effect of PFTs in order to identify the missing links in these pathways. Most of these findings result from investigations in one cell type, while most PFTs intoxicate several. As death programs are cell type-dependent, it would be important to extend these investigations to other cells and tissues. [29,34,55,59]

## 5. Intercellular Junction Disruption

Although not precisely demonstrated, PFTs are probably present at sublytic concentrations in vivo [19]. However, bacterial PFTs trigger additional toxic mechanisms modifying cell behavior.

Cadherins, located in adherens junctions and required for intercellular adhesion, were recently shown to be targeted by PFTs. PFT-triggered cadherin cleavage was first described by Inoshima et al. after incubation of cells with Hla [51]. The mechanism involves the subversion of ADAM10, a transmembrane protease, whose major substrates are adhesive receptors, including E- and VE-cadherins located at epithelial and endothelial intercellular junctions, respectively. In normal settings, when activated by outside-in signals, ADAM10 cleaves cadherin extracellular domains close to the transmembrane region to remove the homophilic adhesive domain (Figure 1). This proteolytic cleavage considerably modifies the cell’s adhesive properties and induces their extrusion from tissues.

ADAM10 is the cellular receptor for Hla. Extracellular Ca^2+^ is required for Hla-dependent cadherin cleavage by ADAM10, suggesting that the Hla-ADAM10 interaction alone cannot activate ADAM10; indeed, Ca^2+^ influx generated by pore formation is involved in this process. A similar effect was confirmed for PLY, which binds cholesterol rather than ADAM10 [51,85,86].

More recently, two PFTs secreted by *Pseudomonas aeruginosa* (ExlA) and *S. marcescens* (ShlA) were also shown to induce rapid E- and VE-cadherin cleavage through ADAM10 activation, even though ADAM10 is not a cellular receptor for these PFTs [48]. In resting conditions, intracellular pro-ADAM10 is bound to calmodulin, preventing its cleavage and activation by furin [87,88]. Both ExlA and ShlA promote a sustained elevation of cytosolic concentration of Ca^2+^ [48], which interacts with high affinity with calmodulin. Once bound to Ca^2+^, calmodulin releases pro-ADAM10, which is in turn activated by furin and transported to the plasma membrane where it induces cadherin shedding (Figure 1). Thus, these PFTs subvert a tightly regulated host pathway, which controls cellular adhesive properties within tissues. Although this effect has only been demonstrated for four PFTs so far [48,51,85,86], it is likely that most PFTs promoting Ca^2+^ influx would induce cadherin cleavage via ADAM10 activation, eventually permitting bacterial transmigration across tissue barriers after complete destruction of intercellular junctions.

ADAM10 substrates also include kinase receptors and matrix proteins (see past reviews [89,90]), which are additional potential targets of PFTs. If confirmed, PFTs might also manipulate signal transduction pathways and the extracellular environment through the same initial mechanism.

Interestingly, tight junctions, which control barrier permeability, are also disrupted by *A. hydrophila* aerolysin via Ca^2+^- and myosin light chain kinase (MLCK)-dependent pathways [28] (Figure 1). This feature further supports the hypothesis that intercellular junctions are one of the main targets of PFTs.

## 6. Other PFT-Mediated Effects

A number of Ca^2+^-dependent effects have been reported in immune cells, including granulocyte chemotaxis [12,40], reactive oxygen species (ROS), cytokine and leukotriene B4 production by granulocytes [40,46,56], cytokine production by macrophages [10], and degranulation and cytokine synthesis in mast cells [9]. Cytokine production is also elicited by PFTs in epithelial cells [39]. All these effects are pro-inflammatory and are expected to promote elimination of bacteria.

In general, erythrocytes are rapidly hemolyzed by PFTs because they have no means of resisting pore formation. Platelets may be activated by pore-induced Ca^2+^ flux, hence providing an explanation for the prothrombotic action of some pathogens [57].

Importantly, pore formation may also facilitate bacterial internalization by triggering a Ca^2+^-dependent protein kinase C-Rac1-Arp2/3 signaling pathway acting on F-actin [43].

In addition to the mechanisms presented above, several intracellular signaling molecules are stimulated by PFTs, such as phospholipase A_2_ (PLA_2_), whose activity is enhanced by Ca^2+^ binding [50], and early growth response protein 1 (EGR-1) transcription factor via the calcineurin-nuclear factor of activated T cells (NFAT) pathway [54]. The final impact of these modifications has yet to be determined.

## 7. Concluding Remarks

Because Ca^2+^ is a very important communicator in cell signaling and drives important cellular functions, its manipulation by bacterial PFTs has profound consequences on cell behavior and homeostasis.

Increased cytosolic Ca^2+^ concentrations can have dramatic consequences, including tissue destruction and bacterial dissemination, or more subtle effects, such as bacterial internalization or thrombosis. However, PFT-induced cell rupture and cytokine production are also alarming signals engaging a strong immune response that counteract the infection.

As indicated above, PFTs from various bacteria may induce diverse—sometimes opposing—effects, however the mechanisms activated by these toxins have not been systematically investigated for all PFTs and much work remains to be done to obtain a general view of the action of PFTs in various infection scenarios.

## Figures and Tables

**Table 1:** PFTs reported to promote increases in intracellular Ca^2+^ concentrations.

Species	Toxin Name ^1^	Pore Size ^2^	Ca^2+^ Origin ^3^	Ca^2+^ Kinetics	Reported Effects of PFT-Induced Ca^2+^ Influx	Refs
*Actinobacillus actinomycetemcomitans*	Ltx	n. d.	EC	Monophasic	∙ Neutrophil lysis	[27]
*Aeromonas hydrophila*	Aerolysin	Small	EC + IC	Multiphasic	∙ Granulocyte chemotaxis ∙ T cell apoptosis ∙ Actomyosin contraction and tight junction disruption	[12,28,29,30]
*Aeromonas sobria*	ASH	Small	EC + IC	Biphasic		[15]
*Bordetella pertussis*	ACT = CyaA	Small	EC	Multiphasic via non-voltage dependent channels with L-type properties	∙ Prevents ACT endocytosis and degradation	[31,32]
*Clostridium perfringens*	PFO	Large	EC	Unknown	∙ Activates/enhances repair mechanism	[33]
	CPE	Small	Unknown	Biphasic	∙ Apoptosis and necrosis through calpain and calmodulin-dependent processes	[34]
	ET	Small	EC	Monophasic		[35,36]
*Clostridium septicum*	α-toxin	Small	EC	Biphasic	∙ Necrosis induced by multiple pathways	[37]
*Escherichia coli*	HlyA	Small	EC	Oscillations due to Ca^2+^ channel activation or to rapid formation/closure of the pore	∙ ROS production by granulocytes ∙ IL-6 and IL-8 production by epithelal cells	[38,39,40]
	ClyA = HlyE	Small	IC	Oscillations		[41]
*Listeria monocytogenes*	LLO	Large	EC IC via G-protein activation-IP3 production	Oscillation due to rapid formation/closure of the pore and release from IC stores	∙ Bacterial internalization ∙ Mast cell degranulation and cytokine synthesis ∙ Immune cell desensitization	[8,9,42,43,44]
*Pasteurella hemolytica*	LKT	n. d.	EC through voltage-gated Ca^2+^ channels	Monophasic	∙ ROS and leukotriene production by neutrophils ∙ Cytokine release from macrophages	[7,10,45,46,47]
*Pseudomonas aeruginosa*	ExlA	Small	EC	Biphasic	∙ Cadherin cleavage via ADAM10 activation ∙ Necrosis	[48]
*Photobacterium damselae*	PhlyP	Small		Monophasic	∙ Lysosomal exocytosis	[18]
*Serratia marcescens*	ShlA	Small	EC	Monophasic	∙ Cadherin cleavage via ADAM10 activation ∙ Necrosis	[48]
*Staphylococcus aureus*	Hla = α-toxin	Small	EC	Monophasic	∙ PLA2 activation ∙ Cadherin cleavage through ADAM10 activation	[49,50,51,52,53]
	Hlg	Small	IC from lysosomes and endoplasmic reticulum EC from store-operated channels	Mono/biphasic		[11,14]
	PVL	Small	As for Hlg	Mono/biphasic		[11,14]
*Streptococcus intermedius*	ILY	Large	Unknown	Unknown	∙ NFAT activation and EGR-1 expression via Ca^2+^/calcineurin pathway ∙ Activation/enhancement of repair mechanism	[33,54]
*Streptococcus pneumoniae*	PLY	Large	EC	Multiphasic	∙ Apoptosis ∙ IL-8 production via NFκB activation ∙ Cadherin cleavage through ADAM10 activation ∙ Activation/enhancement of repair mechanism ∙ Platelet activation ∙ NFκB-dependent IL-8 synthesis	[51,55,56,57,58]
*Streptococcus pyogenes*	SLO	Large	EC + IC	Monophasic	∙ Granulocyte chemotaxis ∙ Keratinocyte apoptosis and ER vacuolation ∙ Membrane repair	[12,33,59,60]

^1^ Ltx, leukotoxin; ASH, *A. sobria* hemolysin; ACT (or CyaA), adenylate cyclase toxin-hemolysin; PFO, perfringolysin O; CPE, *C. perfringens* enterotoxin; ET, epsilon toxin; HlyA, hemolysin-α; ClyA (or HlyE), cytolysin A; LLO, lysteriolysin O; LKT, leukotoxin A; ExlA, exolysin A; PhlyP, phobalysin; ShlA, *Serratia* hemolysin A; HlA, hemolysin-α; Hlg, hemolysin-γ; PVL, Panton–Valentine leukocidin; ILY, intermedilysin; PLY, pneumolysin; SLO, streptolysin O. ^2^ Internal pore diameter. Small: 1–2 nm; Large: up to 30 nm. n. d., not detrmined. ^3^ EC, from the extracellular milieu; IC, from intracellular stores; “Ca^2+^ channels” indicates the activation of cellular Ca^2+^ channels without or in addition to Ca^2+^ influx through the PFT.

**Table 2:** Cellular death programs triggered by PFT-induced Ca^2+^ concentration rise.

Pore-Forming Toxins ^1^ (Species)	Apoptosis	Necrosis	Necroptosis	Ref.
Ltx (*A. actinomycetemcomitans*)	In T cells. Possibly calpain-dependent			[61]
Aerolysin (*A. hydophila*)	At low dose in T cells			[29]
CPE (*C. perfringens*)	At low dose in enterocytes	At high dose in enterocytes		[34]
ET (*C. perfringens*)		In renal collecting duct cells		[35]
α-toxin (*C. septicum*)		In myoblasts		[37]
PLY (*S. pneumoniae*)	In microglial cells		In pneumocytes	[55,83]
SLO (*S. pyogenes*)	At low dose in keratinocytes	At high dose in keratinocytes		[59]
ShlA (*S. marcescens*)			In pneumocytes	[83]

^1^ Abbreviations as in Table 1.
